# Patients as Partners: A Qualitative Study of Patients’ Engagement in Their Health Care

**DOI:** 10.1371/journal.pone.0122499

**Published:** 2015-04-09

**Authors:** Marie-Pascale Pomey, Djahanchah P. Ghadiri, Philippe Karazivan, Nicolas Fernandez, Nathalie Clavel

**Affiliations:** 1 École de santé publique, University of Montreal, Montreal, Quebec, Canada; 2 Department of Management, HEC Montreal, Montreal, Quebec, Canada; 3 Faculty of Medecine, University of Montreal, Montreal, Quebec, Canada; 4 Faculté des sciences de l’éducation, Université du Québec à Montréal, Montreal, Quebec, Canada; Cardiff University, UNITED KINGDOM

## Abstract

To advocate for patients to be more actively involved with the healthcare services they receive, particularly patients living with chronic illness, the Faculty of Medicine of the University of Montreal and its affiliated hospitals developed the Patients as Partners concept where the patient is considered a full-fledged partner of the health care delivery team and the patient’s experiential knowledge is recognized. This study aims to show how patients view their engagement with healthcare professionals regarding their direct care. Using theoretical sampling, 16 semi-structured interviews were conducted with patients with chronic illness who were familiar with the concept of Patients as Partners. Data analysis followed a constructivist grounded theory approach. Patients describe themselves as proactively engaging in three types of practice, regardless of health professionals’ openness to their role as partners. The first is a process of continuous learning that allows them to acquire experiential knowledge about their health, as well as scientific information and technical know-how. The second involves their assessment of the healthcare they receive, in terms of its quality and how it aligns with their personal preferences. It includes their assessment of the quality of their relationship with the health professional and of the latter’s scientific knowledge and technical know-how. The third type, adaptation practices, builds on patients’ learning and assessments to compensate for and adapt to what has been perceived as optimal or non-optimal health or healthcare circumstances. Patients appear to play a more active and less docile role in their own direct care than suggested so far in the literature, regardless of the degree of reciprocity of the partnership or the degree to which the health professional seeks to encourage patient engagement.

## Introduction

Healthcare systems in North America and Europe are currently confronted with the rising incidence of chronic illness [1]. For instance, in Canada it is estimated that one out of two people suffer from at least one chronic illness, and these figures are likely to increase in the coming years [2]. This epidemiological shift is challenging the traditional healthcare delivery models that were developed and established immediately following World War II, largely to provide acute care and manage transitory infectious diseases. Chronically ill patients require long-term follow-up, calling upon multiple professionals who sometimes work in different organizational settings. While the demand for healthcare services is expected to increase sharply [3] as chronic illnesses escalate, it is occurring at a time when financial resources are limited.

The paternalistic approach to healthcare, where health professionals make all of the decisions with little or no input from the patient, has evolved over the past 20 years towards a patient-centered care model [4] that aims to personalize care according to individual patients’ needs, values, and experiences [5]. However, in spite of the undeniable and significant contributions of patients [6], physicians and healthcare professionals retain a predominant role in the healthcare system. Patient participation, or engagement, is only recently receiving greater attention [7] as it becomes increasingly evident that it can be an innovative and viable approach to ensuring appropriate care in the current environment strained by limited resources [8, 9]. Around the world, health organizations, care delivery institutions, and universities are striving to expand patient engagement beyond a token [9] level of involvement.

Recent initiatives, such as shared decision-making interventions [10,11], therapeutic educational approaches [12], increasing patient expertise [13] and self-management [14–16] capacity building, are inspired by patient-centred approaches and point to new ways of integrating patient views [17, 18].

The Patients as Partners (PP) concept emerges from these initiatives and takes patient engagement even further by considering the patient as a full-fledged member of the health care team [19, 20]. Even though there is currently no consensus definition of the concept, it is based on the following assumptions. First, in chronic illness patients are called upon to provide a significant amount of self-care [14–16]. Second, chronically ill patients develop experiential knowledge that can enhance their self-managed care and complement the scientific knowledge of healthcare professionals. In other words, from a patient’s perspective, quality healthcare decisions are based on two complementary forms of knowledge: the scientific knowledge of health professionals and the patient’s own experiential knowledge. Third, such decisions, besides drawing on both types of knowledge, must relate to the patient’s life plan.

For partnership to take place and for professionals to acknowledge patients’ “care provider” status, patients’ experiential knowledge and competencies, developed through their illness experience, must be recognized as a positive and complementary contribution to their health care [21, 22]. This recognition fosters collaboration and the patient’s integration into the interprofessional healthcare team.

This integration hinges on the development and sustained use of competencies and practices by both patients and professionals [23]. Patients make decisions with regard to their own care based on their experiential knowledge, just as healthcare professionals apply their clinical and scientific expertise. Patients’ ability to establish meaningful interactions with professionals depends on their capacity to communicate their experiential knowledge [24].

Around the world, various partnership initiatives have been put into place. In the United Kingdom, the National Health Service has developed a new approach to care incorporating patient expertise. Patients are viewed as experts, entirely capable of managing a large portion of their care [25, 26]. These “Patient Expert Programmes” are aimed at reducing patients’ symptom severity while increasing their confidence, competence, and efficiency in managing their chronic illness [26]. In the United States, the Indiana University School of Medicine Relationship Centered Care Initiative (RCCI) has set a goal to foster a “caring, respectful and collaborative culture.” They have introduced initiatives that shape their informal curriculum to consistently embody and reinforce appropriate professional values with regard to interactions with patients [27]. Similarly, in Canada, the province of British Columbia’s Ministry of Health introduced in 2007 the “Patients as Partners” approach, an initiative with two principal aims: to promote patient involvement and decision-making in the healthcare system by providing patients with relevant tools (competencies and capacity building) and to foster their active involvement in the continuous improvement of quality of care and services [28].

The notion of “patients as partners” has gained popularity over the past four years, particularly at the Faculty of Medicine of the University of Montreal (UM). This faculty currently has a group of patient trainers who take part in the education of students in the health sciences [29] (more than 6,000 so far), ensuring that they are introduced early on to the concept of PP. This program holds considerable promise for sustaining and improving healthcare services for patients with chronic illness. The literature points to the necessity of soliciting and harnessing patient engagement [30, 31] to maintain and enhance the quality of health care delivery in the future. However, clearer conceptual models remain to be developed.

Patient engagement has been defined in various ways [32]. We define it as “the actions people take for their health and to benefit from health care” [7]. Beyond this general definition, the literature on patient engagement points either to patient behaviours that improve health care at various levels [33] or to interventions aimed at engaging patients [32]. Overall, two assumptions appear to underlie the literature on patient engagement. First, that patient engagement includes only those patient activities that are in line with health practitioners’ prescriptions and implicitly excludes attitudes or activities that raise contestation and resistance. In other words, a patient in partnership is often seen as a patient who follows treatments as prescribed and does not challenge healthcare professionals. The second assumption is that the degree of patients’ involvement in their health care is dependent upon the degree of health professionals’ willingness to encourage patient engagement. In other words, patients are portrayed as passive agents who need to be motivated, mobilized, invited, or convinced to be more active and to take part in their own health care.

What is missing from the literature is an empirical study of patients’ perspectives on what they themselves consider to be useful engagement practices for actively enhancing their health care. Particularly relevant in the context of recent initiatives, this more active view of patients as partners calls for further exploration, including description of engagement practices. Hence, the purpose of this paper is to investigate, from patients’ own perspectives, some of the engagement practices that they have put into place in their efforts to achieve optimal health outcomes.

## Materials and Methods

We chose a grounded theory approach [34] for this research, to allow for the emergence of themes revealed by the patients themselves. Grounded theory does not allow for initial hypothesizing; however, there are certain *a priori* assumptions that influenced our data collection, analysis and interpretation. Our main assumption is that patient partnership leads to positive outcomes for patients, first, on a personal level, and second, because of the relationship that develops with healthcare professionals. Precautions taken to mitigate the impact of these assumptions at all stages of the study included keeping logbooks, engaging in frequent reflexive discussions among the authors, and systematically seeking divergent opinions and probing for negative effects on health outcomes.

### Study Design

The theoretical sampling approach [35, 36] was used for participant selection and recruitment. This approach rests on the assumption that “intense cases” are appropriate or successful examples of the object of study, containing rich information about the phenomenon, i.e., in this instance cases involving participants familiar with and receptive to the concept of PP. Study participants were recruited from among patient trainers at the Faculty of Medicine of the University of Montreal. Since 2010, these trainers have been co-leading, with clinicians, interprofessional collaboration courses in 13 health sciences training programs. By sharing their experiences of interacting with the healthcare system, they ensure students become aware of the importance of Patient Partnership [37]. For this study, participants had to have participated in at least one interprofessional collaboration course at UM during the last year and have completed a training course on the concepts of partnership of care [23]. Since participants were selected because of their familiarity with the concepts, they were able to talk about them in relation to their own experience of care. Although many had managed to establish successful PP relationships, this was not a selection requirement for the study. Our goal in recruiting participants was for them to inform us generally about practices of patients living with a chronic condition, not just about PP practices. Indeed, most of the participants were ill and engaged in their own care process long before they were sensitized to the concept of Patients as Partners. They also reported that the majority of their experience had not been “in partnership,” and that this was precisely one of the reasons they had become involved in the Patients as Partners approach.

Qualitative sampling requires that sufficient data be generated to adequately explore the phenomenon under investigation. Our sampling strategy was designed to allow us to explore perceptions both of patients living with at least one chronic illness and of caregivers of such patients, while ensuring they were able to speak reflexively about their experience and relate it to the concept of Patients as Partners (PP).

### Data Collection

Potential participants were identified by the UM Faculty Collaboration and Patient Partnership Unit (CPPU). The CPPU provided a list of 18 potential participants. Of these, 16 were willing to be contacted, and all 16 agreed to participate in the study. The research assistant contacted participants by telephone to confirm their interest, screen for eligibility, and arrange for an interview. They were eligible for participation if they had at least one chronic illness or were caregivers for someone with such an illness.

Interviews were semi-structured (see the Participant Interview Guide in Appendix 1). The interview guide was tested to confirm that the length and flow of the questioning were appropriate. All questions were designed to be exploratory, to allow multiple perspectives of participants’ perceptions and experiences to emerge during the course of the interview. Specific focused themes, such as living with illness and with care, relationships with healthcare professionals, self-reflection mechanisms, etc., were explored with all participants. An inductive interviewing approach encouraged subjects to express themselves freely, thereby allowing themes to emerge.

Interviews took place between July 2013 and June 2014 on UM premises. All interviews were conducted by at least two authors experienced in qualitative research. Interviews ranged in length from 60 to 90 minutes. We determined that theoretical saturation was achieved after 10 interviews (out of a total of 16), as analysis of the six subsequent interviews did not generate any further leads but rather confirmed the stability of our theoretical categories and their properties (Glaser & Strauss, 1967).

In total, 16 participants (10 female, 6 male) were included in this analysis, to achieve saturation [38]. Participants’ ages ranged from 22 to 53 years, and the number of years living with a chronic illness ranged from 1.5 to 43 (see Table 1).

**Table 1:** Study Participant Characteristics.

Pseudonym	Age	Sex	Illness	Number of years with illness
**Orlando**	46	M	Chronic kidney deficiency	23
**Donna**	29	F	Lupus	4
**Naomi**	45	F	Arythmogenic displasia of the right ventricle	7
**Norman**	55	M	Type 1 diabetes	43
**Denise**	50	F	Mother of child diagnosed at birth with development trouble	
**Harry**	22	M	Crohn’s disease	4
**Trisha**	46	F	Multiple sclerosis (MS)	17
**Karen**	35	F	Mental illness	17
**Marie**	53	F	von Hippel-Lindau (VHL) disease	8
**Frank**	43	M	Dyslipidemia	6
**Laurence**	48	F	Algodystrophy of the shoulder	20
			Sciatica	3
**Vera**	35	F	Multiple sclerosis (MS)	10
**Victor**	65	M	Type 2 diabetes	10
			Parkinson’s disease	1,5
			Caretaker relative of a patient diagnosed with cancer	5
**Chloé**	31	F	Type 1 diabetes	17
**Paul**	66	M	Tonsil cancer	5
**Josée**	68	F	Breast cancer	1.5

Each participant was given a pseudonym in order to ensure confidentiality and anonymity.

### Data Analysis

We used constructivist grounded theory approach for data analysis [34]. This approach provides evidence pertaining to the phenomenon by theorizing that people assign subjective meaning to their everyday experiences. Like most qualitative analysis methods, grounded theory is based on the concept of emergent themes. These themes are used not only to explore an issue, but also to construct a cohesive idea or theory about an investigated phenomenon as it emerges from the collected data [39].

Each interview was digitally recorded and transcribed; none were returned to participants, as all recordings were clearly audible. Transcripts were imported into NVivo 9 for Windows (QSR International) for data coding. In compliance with criteria for methodological rigour in qualitative research [36, 40], two techniques were used for coding: primary open coding, followed by thematic and selective coding. Data analysis was carried out concurrently with data collection, as per the grounded theory approach [39]. Two authors coded the first three interviews independently. After the first phase of primary coding, the group discussed the data and reached consensus on an initial coding tree. Coding categories were discussed and agreed to by all authors; divergent issues were discussed until the group reached agreement.

In a second phase of thematic coding, links between different codes were analyzed and discussed to create a thematic coding structure. Through a constant comparative process [34], selective coding was used to generate and refine categories (coding groups), leading to a conceptualization of the phenomenon under study.

After five interviews, concepts and categories were applied to all interview transcripts. Coding categories were subsequently populated with quotes to ensure grounding of the data and representation across the study sample, thereby providing an integrated account of participants’ practices and how they view PP.

Careful attention was paid to the use of language in participants’ accounts, as well as in naming the codes. Regular meetings provided opportunities for reflexive sharing as the research team considered how their assumptions and beliefs might impact data interpretation. The goal was not to attempt to establish the “truth” in participants’ accounts as measured against an objective reality, but rather to discern the meaning they assigned to their experiences. Finally, a logbook was kept to ensure reproducibility of the analytic procedures. Summaries were drafted after each interview, and memos after each research team meeting.

We presented our findings at a colloquium in Montreal in May 2014, as well as at a CPPU meeting in September 2014 where patients and carers from the CPPU and other organizations were present and able to comment on our research findings. They unanimously confirmed that our findings matched their understanding of how they behaved with their healthcare professionals [41].

### Ethical Considerations

Ethical approval was obtained from the UM Committee for Ethical Research in Health Sciences (Certificate #13036-CERES-D), and each participant gave informed written consent at the start of the study.

## Results

We organized our findings around three main types of engagement practices that emerged from patient interview data: 1) learning practices, 2) assessment practices, and 3) adaptation practices.

### Learning Practices

According to our respondents, they constantly engage in learning practices. Being ill, for them, is a learning process. The learning practices they mentioned included collecting scientific and medical knowledge about their state of health by familiarizing themselves with relevant medical vocabulary and procedures, acquiring medical technical know-how, and developing an understanding of organizational constraints associated with treatment of their condition. Moreover, they discussed the importance of knowing themselves, in terms of how they live with their illness and its treatment. Indeed, over time chronically ill patients and their carers can develop extensive knowledge concerning the symptoms that they experience and the effects of different interventions on their state of health and on their lives:

Learning about the symptoms of your illness is all part of the process, if it’s something serious or not. If my bowels start grumbling, I know if it’s serious or not. (Harry)

Let me give you a concrete example. I’ve been a diabetic for a long time so of course I know how much insulin I need. I know that if I am physically active, I have to take less; if I eat certain things, I have to give myself a little more. (Norman)

I want to know everything. What’s this, what’s that? What are you doing? Why this? Why that? What are the side effects? How do the different medications interact? It’s… I want to know everything. (Chloé)

Patients may also devote considerable effort to mastering scientific knowledge about their illness. Some of them try to be proficient in the medical terminology of their illness, while others also undertake in-depth reviews of scientific literature:

Internet. Let’s say I started by learning words. Because you see, well… medical words, if you don’t have to deal with those words, you have no clue. This helped me, the meaning of words. Anyway, I don’t only do it for medical reasons. (…) And… I can now function with these words. (Paul)

I did some research on my illness. I saw that there were really no medications for me that I could take to change its progression. (Trisha)

I know about my illness, of course. I know a lot about my illness, more than a lot of doctors because it isn’t possible for them to study this illness and keep up with all the conferences and all the articles that are published all over the world…. I can describe it really well. I can tell you about the development of different tumours and when they become hemangiomas. My vocabulary is richer because of all the reading I do about it. But at the same time, I tell them: it’s because I’m interested in what I’m going through! (Marie)

In some instances, this scientific knowledge can be obtained directly from the health professionals they meet. Patients described themselves as proactive in the learning process. This might involve asking questions, as Frank recalls:

I ask questions. I’m the kind of person who likes to know, I’m interested in what people do, I pay attention to what they say. I also like to know what’s happening to me. It’s by asking questions to health care providers that I gained this knowledge. I did this with my cardiologist, with the nutritionists, with the other cardiologists I saw. (Frank)

This process also implies expectations regarding, as one of the respondents put it, “the teaching that can be done by the care provider” (Harry), namely that care providers will provide proper information.

Patients and their relatives also learn about themselves, in terms of their potential roles, limits, and possibilities as partners in the healthcare relationship:

A while ago, I was telling you about accompanying my wife through terminal cancer. Well, I did go with her to all her appointments because she was in denial, that is to say she would only listen to what she wanted to hear and she would lose strands of what she was told, so I took notes of everything so that I could help her better and know what the exact situation was. So, having learned from this experience, I tell myself that sometimes, in unusually important matters, serious matters, it’s important that… that there must be someone else who can corroborate or maybe hear parts of what is being told that I myself could miss. (Victor)

Some participants learn specific care techniques or procedures:

The dermatologists had been treating the feet of diabetics for years and years, and they had shown me how to apply the dressing. They even showed my wife, who was always with me. She could see how it was done. (Norman)

If you have to learn how to inject yourself, you learn and you inject yourself. (Donna)

The peritoneal dialysis went well and for a week I was being taught. I would go to the hospital every day. There was a nurse and there were two or three other patients. They showed us how to seal the tubing so there is no leaking, how to wash our hands, how to prepare the puncture site in order to avoid contamination. They were really teaching us! (Orlando)

Learning practices sometimes involve gaining a greater understanding of how health care delivery is organized, as well as an appreciation of the systemic or institutional constraints that health professionals deal with in their work. Karen comments on what she understands she can expect from her health practitioners:

They have managers who put them under a lot of pressure. Access to mental health services is through a single window system and people are entitled to eight treatment sessions, no matter what their health problem is. Someone shows up with a bipolar condition, who is diagnosed with the illness for the first time—he is entitled to eight weeks. It’s obvious that no health professional will go along with that! They will find a way to get around it, but even so, there is still that indicator and we have to make do with it. (Karen)

In summary, respondents revealed how they are constantly learning, acquiring not only experiential knowledge about their illness and its treatment, but also other types of knowledge, whether scientific, technical, or organizational, that are generally considered to be the prerogative of health professionals.

### Assessment Practices

Patients continually assess the quality and appropriateness of their healthcare professionals’ interventions, of their medical knowledge and people skills, and of the relationship that develops between them.

They assess the effect of treatments not only on their health condition, but sometimes also, as in the case of Trisha, on their personal life:

It’s just that there were (…) protocols and things like that, and I realized that they took too much of my time and I wanted to live my life, so with most efforts it was like, “How can I both live with what I have and take advice from doctors and health professionals?” (Trisha)

They usually assess clinical procedures, whether negatively or positively, in terms of consistency, security, and quality.

I was VRE positive for a while. I saw how flawed hospital protocols are. On the one hand, people have to put on specific uniforms before entering the room and, on the other, the cleaner comes in to empty the waste bin without having to wear the uniform. The doctor also comes in without changing. There are lot of inconsistencies in these protocols. (Frank)

I had been to see a clinician who was really good. He was at the R… Hospital. These people had been caring for diabetic feet for years…. And when I would go to the community health services center, they would do my dressings, but they would do them all wrong. (Norman)

Patients also assess health professionals’ knowledge about their illness and compare it with what they have learned:

I have a lot of problems with the nutritionists! (Laughter) I find that what they recommend is never suited to people with Crohn’s disease; they know nothing about it. When I tell them, for example, that raw vegetables are out of the question, except for carrots and mushrooms, they seem troubled and they don’t know what to do, so much so that the conversation often ends there. (Frank)

I noticed that, at the clinic, they didn’t know how important foot care is for diabetics. (Norman)

Another aspect of the assessment focuses on the quality of the relationship that is established with the healthcare professional. Again, it can be positive or negative. In the following excerpt, Donna assesses her relationship with her doctor:

I thought that it was very respectful, on his part, to fall back on his own humanity and to tell me the truth… you know, instead of…. He came to see me all by himself, after he had made all his calls, he came to chat and said: “I don’t know what you have,” and we talked and talked. You know, we comforted each other and we talked. I found that… I am an atheist, I don’t care much for spirituality, but I found myself alongside a fellow human being. We are both going to die some day, we both have to face our fears at some point, and there we are… just sitting and talking. (Donna)

Donna assessed the relationship in terms of respect, transparency (telling the truth, even about the limits of his expertise), closeness (“he came to chat”), equality (“a fellow human being”; “we both…”), and mutual concern (“we comforted each other”), as opposed to an idea of the doctor as a remote, distant, or patronizing figure engaging in controlled and insensitive interactions.

Denise, a patient and a carer for her son, assesses her relationship with her doctor in terms of respect for her point of view:

When I say “my coach,” it’s true! She was much more than a resource person. She took me into consideration, she gave me all the space I needed. I would speak for my son. M. wasn’t able to express himself! So everything went through me, and the doctor always accepted what I accepted and what I said as being true. She didn’t diminish what I was saying, she always took into consideration my point of view. (Denise)

In this extract, Denise portrays her son’s doctor foremost as a coach who respects her needs, her views, and her decisions. As in Donna’s assessment, the power relation is represented in more egalitarian terms; the distance to power is lessened, and the patient’s experience, knowledge, and decisions are not discarded or treated with condescension.

Laurence’s positive assessment focuses on her doctor’s mindful presence with his patient:

My GP (…) would ask me questions from an overall point of view, not only about the pain, because he could see me, he was interested in me. And also what I liked was to see that he loved his work, he seemed happy, he didn’t seem stressed out, he took his time with me, I could see that he was in no rush, although I understand that he could be under quite a bit of pressure. But when I was with him, I really felt that I was with him. (Laurence)

Laurence assesses the relationship in terms of the quality of the health professional’s presence (“I really felt I was with him”). For her, taking time, taking a real interest in the patient, and not appearing rushed or pressured to move on to the next patient were indicative of the quality of the doctor’s relationship, not only to his patient, but also to his job.

On the other hand, some patients assess their healthcare professionals negatively. In the following quotation, Chloé compares doctors’ attitudes:

I really appreciated the doctors who told me, “I don’t know what you have. But we’ll figure it out together.” That’s really incredible. But when they say, “You’re imagining things, it’s all in your head. There’s nothing wrong with you.” That is incredible, too, but in a bad sense. (Chloé)

Chloé presents the stark contrast between doctors who are transparent about the limits of their knowledge and who engage the patient as a partner (“We’ll figure it out together”), and those who not only discount her point of view but also deny her experience of feeling that something is wrong.

In the following extract, Vera recalls her doctor’s lack of compassion at a crucial moment in her healthcare trajectory:

When he gave me the diagnosis, he was reading his notes. And I still remember it, though it was almost ten years ago. You already don’t feel great. I already knew, I knew that it was MS. You already don’t feel great knowing that your GP is going to tell you that you’ve got MS, and then when he tells you, he’s reading his notes, not even looking you in the eyes, it takes something away … You know, you just need a bit of … I’d say a bit of compassion, of comforting. I know it’s not the doctor’s job to do that, but you are learning some news that’s going to change the rest of your life. (Vera)

Although she mitigates her assessment of the relationship by telling herself and the interviewers that she “knows” it is not a doctor’s job to have compassion, the rest of her quotation says otherwise, pointing to the lack of eye contact as symbolic of the lack of comfort she experienced at a time when she needed it the most.

The next quotation, from Naomi, again stresses the importance of the healthcare professional’s being present, listening to the patient’s words, and looking the patient in the eye:

The things you feel… that’s also when you realize that they aren’t listening to you, that they didn’t hear you, that you would have needed them to look into your eyes a little bit. Sometimes, that’s all there is to being a patient partner, you just want to feel that you are a partner, a part of the treatment plan; you just want the doctor to put down his pen and look at you… for a few seconds! (Naomi)

For Naomi, it all boils down to whether or not patients feel treated as partners in their health care.

Yet in their assessment of health professionals, some patients, such as Paul, tend to mitigate the potential harshness of their assessment by putting health professionals’ shortcomings in perspective.

Of course you’ll hear, like, “Hey, did you ever look stupid!” Yeah… but she could have been there for ten hours, that lady, the nurse (…) So you might have to take it easy, give her time to take a breath too. (Paul)

Patients also assess themselves and their families and friends in terms of the quality of the role they can play in enhancing their health care:

Vera: The patient and the family also, it’s very important. Because the patient might not be able to talk or, I don’t know, to tell all the different events. On the other hand, the partner who lives with the patient may have some things to tell the health professional that could be helpful.

Interviewer: Was that your experience? Your family was very well integrated into the team?

Vera: Very much so. Because I don’t talk much. I wouldn’t speak at all because I was having trouble and I was embarrassed by the state I was in. Also, I had a roommate at the time who was my mother’s age, so she helped me a lot with this, talking about my situation at home (…). Because as a matter of fact, at that point in time I wouldn’t have been inclined to ask questions to the professionals, to my neurologist, to anyone, in fact. But she did ask, and they were always the right questions. (…) Because at that stage I wasn’t ready to ask my mother to come with me because my mother had completely fallen apart.

In the above excerpt from her interview, when Vera notes her unwillingness to speak, she not only assesses her ability to fully act as a partner in her health care, but also assesses the actual ability of those close to her (her roommate and her mother) to compensate for her shortcomings as a partner.

In summary, patients’ critical assessments focus not only on the relevance and appropriateness of health professionals’ recommendations and actions, but also on the quality of their interactions and of the relationship. Moreover, patients assess not only the role played by their health professionals, but also the role played by their relatives and themselves in managing their health care.

### Adaptation Practices

The third theme that emerged from our analysis centred on adaptation practices that patients enact to enhance their health care. Building on their learning and assessments, patients use various strategies to fill the gap between what they perceive as non-optimal health or healthcare circumstances and their healthcare goals and needs.

In the following excerpt Trisha describes how she engages in her own health care through daily life adaptations to her condition that compensate for her own limitations.

I mean, for me it was something that I call “adaptism” because it’s a life of adapting. You have a progressive chronic disease, what else are you going to do? So I thought instead of excluding it from my real life, from my life, you know, I’ll incorporate it and even brushing my teeth, in the beginning, when it was really hard for me standing, I would stand up. (Trisha)

Our respondents’ testimonies reveal that adaptation practices may also extend beyond the patient’s domain into that of the health professional. One example is Naomi’s assessment, where she asserts her opinions and preferences, even to the point of openly contesting the doctor’s prescribed treatment:

I remember feeling desperate and telling the doctor: “I don’t want these shocks from my defibrillator anymore.” And the doctor I was seeing then would say: “You have to learn to live with the shocks.” And he would say: “I’ll change your medication….” I was taking amiodarone at the time and I would tell the doctor that it wasn’t working. I couldn’t even cross the street. I felt crushed, frail. I was afraid of everything and I was suffering from arrhythmia all the time on top of that! Things weren’t going well at all. And I would say: “Your medications aren’t working,” and he would answer: “Well, one day I’ll change them.”… He didn’t want to give me other medication and he wanted me to learn to live with the shocks. I don’t think a well-balanced human being can live with shocks. (Naomi)

Thus, adaptation practices include patients voicing their concerns about elements they perceive as lacking and explicitly formulating what they see as solutions to compensate for these deficiencies.

When it was administered intravenously, I would absorb it; when it was administered orally, I didn’t absorb it. So after two or three weeks, I would collapse and go back to the hospital. So I told the attending physician: “Listen, is there a way that I can learn to inject myself?” Because I could see that there were other alternatives than taking cortisone orally…. I know that the medication works when I am in the hospital and I know that it doesn’t work when I’m at home. (Donna)

I would sometimes say to the nurse: “If that’s all the insulin you’re going to give me, my sugar level will be very high!” and she would answer: “No, no, that’s the protocol, that’s what is written down!” I would say: “I’m telling you, if you don’t listen to me…” She would come back and I was hyperglycemic: “Wow… that’s too high, sir!” “I told you, I know myself!” Later on, there was a woman doctor, she understood, she came to see me and said: “N., we’ll change this. You’ll take the insulin that you need to take, you’ll tell the nurse how much that is and she’ll follow the doses you say you need. If we see that it’s really not working, we’ll step in to take control, but…” In fact, I was the one who took control. (Norman)

Patients sometimes react to the extent that they take charge of certain health care activities to accommodate their personal preferences or to mitigate the deficiencies of the health professionals. In certain cases this is done with the health practitioner’s knowledge and agreement, but in others it is done without sanction. Some examples can be seen in the following excerpts:

Finally, when they told me: “We’re putting him in intermediate care,” I said: “In that case, can I go home? I don’t need to wait until he goes into regular care, I’ll do everything through outpatient care. I’ll come every day if necessary, etc.” And they said: “OK.” (Denise)

Once we would get back home, most of the time, my wife had to do the bandage over again because it hadn’t been done properly. So, we said: “No, this is what we’re going to do, if you don’t mind.” I said: “Give me the supplies and we’ll take care of that from now on.” (Norman)

These excerpts illustrate adaptation practices that can sometimes lead to outright contradiction of health professionals’ advice. But adaptation practices do not always entail confrontation and polarization. Some are complementary, such as patients’ proactively preparing questions for medical appointments that might otherwise lack structure or bringing up relevant medical knowledge to counterbalance possible gaps in the health practitioner’s knowledge. Adaptation practices can also mean maintaining health care continuity by taking notes during the consultation so that neither the patient nor the health professional forgets what is said from one meeting to the next.

I was really prepared. I write down everything they tell me because I know very well that we forget as soon as we are out the door…. On top of that, all my questions, I had written them down in advance…. I structure the meeting myself, but that’s the problem, we have no information about what they’re thinking. There is no interaction…. Every idea about possible treatments, including shock therapy, most of the time, I was the one who brought them up after reading the literature at home. (Karen)

Patients also adapt to what they see as their own limitations, by drawing not only on techniques but also on relatives whom they know can compensate for their limitations:

I make a list, sometimes. Because often we’re a bit nervous when we go to see the doctor because we don’t feel well and we’re afraid we’ll forget. So I make a list of questions. A lot of times I’ll even call my brother or my friends: “What should I ask? Have you got any ideas?” Because otherwise I’ll come out and then, “Oh, shoot, I should have asked that!” Yes, it is important to be prepared because it never fails, we’ll forget something. (Laurence)

In the same vein, patients also spoke of the importance of implementing practices that ensure continuity of care, including making appointments for tests to be done and for follow-up:

As for the other problems I had, none of the doctors had followed up. It seemed to slip between the cracks. I was the one who called the genetics specialist to verify that the follow-up was being done, because no one else had done it…. No one takes care of that and says that in “x” months you have to get an eye exam. I had to coordinate those things. (Marie)

For instance, it was extremely complicated to get electroshock therapy because there are only two places in Montreal that offer this treatment; otherwise you have to go to Quebec City…. It took months for the medical team to make the calls. Finally I was the one who got the number of the person in charge here in Montreal. I made all the arrangements to get the appointments. It was really far. It isn’t easy to do this, especially in the state you’re in at that time. (Karen)

So then, I am the one who has to contact the genetics specialist to make sure that there is a follow-up, because no one takes charge of the situation and says: “OK, M. is due for her eye exam, it’s been six months or it’s been a year.” I’m the one who has to coordinate these things. (Karen)

Marie underline the importance of adopting certain daily practices and explain how this contributes directly to maintaining the quality of their health. Such actions complement and augment the interventions of health professionals. Sometimes, as in the following excerpts, health professionals welcome such initiatives:

[They said:] “Mrs T., you’ve been a good patient because when we would suggest you do some activity with us here, you would also do it when you got home, even if we didn’t tell you to.” I said: “It’s because you do things for me, you’re working to help me! It’s up to me to help you help me!” That’s how I see the path I need to follow. (Marie)

In other cases, healthcare professionals are less open to patients’ concerns and initiatives. Some patients report that they make efforts to compensate when they do not see their relationship with the health practitioner as optimal. This can include developing strategies to get their health professional’s attention, as in Marie’s case:

So sometimes I see he has to hurry today. I meet the onco-urologist and I see he’s in a hurry. So I look in my tool box and I am able to read Dr. K’s body language. And then I say to myself: “Just say what you have to say, take your time and tell Dr. K: ‘I know you’re in a hurry, but I’ll start to cry if you don’t let me speak!’” So then he says: “Oh, sorry, you’re right.” He says: “I’m sorry. Things are crazy today.”… But I think a good patient has to feel at ease with his doctors to be able to tell them how they’re feeling and not tell lies. (Marie)

Along with these more confrontational adaptation practices, patients also reported that in certain conditions they prefer to put themselves completely in the hands of the professionals. When the illness is active, intense, painful, and overwhelming, they are ready to relinquish their autonomy and rely on their health professionals’ competence without any restrictions. In other words, in certain instances they consider being taken care of fully as the best option for optimizing their health care:

And then they told me: “Listen, you can’t go on with peritoneal dialysis, you really need to switch to dialysis, to hemodialysis.” It all happened so fast, I was in a daze. I had reached the point where I was ready to abandon everything, a bit like being on holiday—you don’t have to worry about anything anymore. Someone else will take care of you. So, I accepted the things they were telling me, they put me on a gurney and wheeled me off to radiology. (Orlando)

I just couldn’t think about anything else but my illness, to the point where I couldn’t manage anymore. I was in a state of crisis. I was at home and I was anxious about it all. I couldn’t let five minutes go by without thinking about it, without worrying about this or that symptom …. It’s just that they operated on me Wednesday instead of Thursday, because it was no longer manageable, to some extent …. And because these are the moments when you say to yourself, as a patient: “I’ll rely on the knowledge that these people have, they were trained to care for people.” At some point you have to be able to say to yourself: “OK, I don’t understand what’s happening anymore, I don’t understand anything.” At some point, we can’t have an objective view of the situation. I think that at that point it’s time to let someone else make the decisions, in order for everything to really get better. (Harry)

Another adaptation practice, adopted by some patients who were not satisfied with their health care situation, consisted in seeking out a different health professional who could better respond to their specific needs or help them achieve their goals:

I was angry when I saw the way they were treating my foot. Of course, it angered me. This is one of the reasons why my dermatologist… when I saw that he wasn’t the right person anymore… I said to myself: “I have to find someone who will be able to take this into account.” (Norman)

First, he sat down and he looked at me. I felt a connection between us and I thought that he would listen to me. Then he checked the defibrillator and he said to me: “You’re right. This is wrong. You can’t live like that.” Obviously, I couldn’t live like that. And at that moment, by the end of the appointment, I felt hopeful and I looked at the doctor and I asked him: “Can a patient shop around for a doctor?” I’m not going to tell you what he said, but we both laughed. Then he said: “You can choose a husband if you want.” We both laughed at that. Then he said: “Yes, you can shop around for a doctor.” I said: “So today I choose you as my doctor.” And he shook my hand and, at that moment, I felt we were a team. (Naomi)

As some patients search for what they perceive as the most appropriate health professionals for their condition, they may also develop strategies to enhance their chances of getting access to specialists, as illustrated in the following excerpt:

When I found out I had diabetes, I registered for a course at the clinic (…) where they were doing research. Yes, I had a plan in the back of my mind; I told myself, “I’m going to meet a specialist who will follow my case and that will make it easy.” In the end I was right. (Victor)

Some adaptation practices may be pursued for the benefit of the individual patient, while others appear to be pursued for the benefit of a community of patients. Indeed, sometimes adaptation practices translate into helping other patients. Having experienced their own difficulties, some patients seek ways to help others who might be undergoing similar circumstances. As Donna recalls:

I wanted help and no one wanted to give me any. I wanted medication to change my situation and no one was trying anything. Then I said to myself: “I’m going to help others, because I went through all that.” I discovered the silver lining in helping others. We all live through the same things in the end! (Donna)

Patients adopt various strategies to bridge the gap between their assessment of their situation and their health care needs and goals. These range from questioning a healthcare professional’s decision to disregarding it, with or without the professional’s acknowledgement. Other strategies include patients’ taking responsibility for scheduling their follow-up appointments or even finding other professionals more inclined to institute a patient partnership relationship.

## Discussion

The aim of this study was to gain a deeper understanding of the experience of living with chronic illness from the perspective of patients who are sensitized to the concept of Patients as Partners and to explore the kinds of practices these patients engage in to enhance their health situation.

Drawing on the results of our interviews, we have highlighted three categories of patient engagement practices that respondents said they used to enhance health care outcomes: learning, assessment, and adaptation. These three categories of engagement practices are closely related and allow patients to enhance outcomes, even when healthcare professionals show limited openness to establishing a partnership or to supporting such practices. Thus, some patients adapt to situations that are less than optimal by using their knowledge and assessment of the situation. This result corroborates Edwards and colleagues’ [42] finding that “patients with a long-term condition can develop health literacy skills over time and put their skills into practice in becoming more active in healthcare consultations.”

The following dialogue with Josée illustrates the close interrelationship between these three categories of practices, with the assessment happening first, followed by (collective) learning and adaptation:

Josee: There are two of them. For routine appointments, we show up and sign in. And at first, I didn’t know. So we’d either see one or the other. And I often saw the other. Until I realized how it worked. Like, everyone would show up and say, “I want to see the nice one.” That’s how I’ll say it so that I don’t have to name names.

Interviewer: OK, so you weren’t the only one to like him better?

Josee: No! For sure. It’s a well known fact, well known! So now I never see him anymore because I always ask for the nice one (…). So you just have to know how the system works. So you say … basically, what is it that all women do, eh? All the women that are being treated, what do we do? We spread the word amongst ourselves. “Well, just ask for … ask for the nice one.”

Patients sometimes use these practices simultaneously. They may also use them to varying degrees, depending upon the current, ever-changing situation: in their lives, with their chronic conditions, and in their relationships with their healthcare professionals.

This study makes several contributions to our conceptualization of Patients as Partners and to our understanding of patient engagement practices. First, our results document the engagement practices used by patients who are sensitized to the idea of partnership. Second, we question the assumption that patients’ degree of activity in enhancing their health care is dependent on health professionals’ willingness and efforts to engage them [32]. We show that some patients can play an active role no matter how open the health professionals are to their involvement. Moreover, we discredit the assumption that engagement practices are necessarily practices of consent to health professionals’ prescriptions [43]. The patients we interviewed were ill long before they considered themselves as partners in care. In other words, these practices can be seen more as inherent in the condition of living with at least one chronic illness than they are the product of being involved in CPPU activities. Based on our respondents’ perspectives, engagement practices can also take the form of contestation and resistance to healthcare professionals’ prescriptions. This study contributes not only to the literature on patient engagement, but also to the conceptualization of patient partnership in the literature, which is based on three principles [19, 20, 29, 37, 44]: 1) recognition of the patient’s experiential knowledge; 2) the patient’s capacity to become a full-fledged member of a healthcare team; and 3) alignment of healthcare decision-making with the patient’s life plan. While this definition is a valid one, our research shows that some patients do not wait until they are placed in this type of interaction to take an active role as a partner in improving their care. Thus, an original contribution of our research is that it broadens the range of what we consider healthcare partnership practices to include practices enacted in situations where the health professional does not reciprocate or where patient engagement methods have not been implemented. We argue that some patients can also act in ways that provide for their own health care, i.e., as active agents who take steps to improve the quality of their care, whether or not there is a reciprocal partnership with a health professional. In addition to any methods put into practice by health professionals to engage patients [32, 45], our study shows that some chronically ill patients will take the initiative of engaging in practices to enhance their health care.

As a result, we also submit that there are different levels of reciprocity in partnerships, from fully reciprocal partnerships to their opposite extreme, situations where patients take part in improving their health care without being acknowledged as partners and without their learning, assessment, or adaptation practices being recognized.

### Implications

Our findings suggest a new way of perceiving the role of patients and their ability to engage in their own care as partners, depending on the degree of reciprocity in the partnership; Patients as Partners are active rather than passive agents, as was succinctly observed by one of our respondents:

The patient must do his part, as well. He can’t just expect his health professional to do everything for him. I can understand that health professionals are overwhelmed, I can see it because I work in that same environment. The patient has a job to do, too. (Vera)

Patients’ roles in healthcare partnerships, as well as the specific engagement practices that they will enact according to the level of reciprocity in those partnerships, require further investigation. In addition, future research should explore the engagement practices of patients who are not familiar with the concept of Patients as Partners.

We can also assert based on our findings that patients may become active partners even when conditions for partnership are not met. Some patients understand that they can not only participate in managing their own health and health care, but also actively establish partnerships with healthcare professionals who are open to the possibility.

This research enriches our understanding of the practices available to patients and is likely to have a direct impact on the way health professionals perceive their relationships with patients. Health professionals are often ill at ease when patients bring up certain information or assess the treatment they receive. Yet such behaviour is an integral part of what patient partners consider normal in the course of a doctor-patient relationship, perhaps even more so in today’s context, where an increasing number of patients have greater access to information about both their illnesses and their treatments through the Internet. Indeed, a recent study revealed that 80% of Internet users occasionally or regularly seek health-related information [46]. Some patients are capable of forming their own opinions about what kind of health care they need [47]. By being mindful of this, professionals will be more inclined to see these practices as enabling a partnership and less inclined to see them as attempts to unmask their professional weaknesses or to undermine their authority. Moreover, knowing that some patients actively adapt in order to enhance their health may help health professionals to better understand these patients’ behaviours. This, in turn, can help the professionals to better inform patients about risks associated with certain adaptation practices (e.g., self-care, non-compliance, etc.), thereby enhancing the reciprocal partnership. To implement engagement methods, it is important to take into consideration patients’ own engagement practices and to tap into any potential synergy.

Therefore, it appears that it is not incumbent on health professionals alone to “carve out a place” for patients and find ways to ensure patient engagement; neither need professionals consider it their sole duty to place patients at the centre of their work. Patient partners do their part by acquiring appropriate knowledge and developing engagement practices such as those observed in this study. It is not only a question of being selective and using what works best; partnerships can ensure a clear focus on optimal health care outcomes. As our respondents informed us, being a patient can involve learning, assessing, and adapting to the idea of achieving better health outcomes.

Our findings will also have an impact on the training of health professionals. It is hoped that health professionals will be better prepared to recognize and encourage various engagement practices that patients might use to improve their health care. This could include raising awareness among patients with chronic illness and training them in a range of possible patient partner practices. In such a context, it would be advisable to establish an educational framework and point out potential risks associated with certain engagement practices, depending on the type of chronic illness, such as the risks associated with certain self-directed care practices that patients sometimes turn to when they are not being treated as partners. Since not all patient engagement practices are necessarily beneficial to patients’ health, research on the effects of such practices on health care outcomes is required.

Furthermore, these results are likely to have an impact on organizational practices in health institutions as well as on healthcare policy. It is important that administrators and managers consider this view of patient partnership, to help health professionals and patients develop successful partnerships that include patient-applied practices. Our results will also have an impact on policy-makers, who must be made aware that some patients acquire important knowledge, need to assess their interactions with health professionals, and are able to adapt to their illness, and that government policies should take them into account. Finally, these results can afford a deeper understanding of what Wagner [48] describes as productive interactions between patients and their families, and healthcare professional teams.

### Study Limitations

People who consider themselves “intense” examples of the study phenomenon because of their experience of living with a chronic condition afford a relevant view of the phenomenon. It is safe to assume that interviewees were referring to common conceptual constructs.

The first limitation of this study is that it focuses exclusively on the patient’s point of view. To gain greater understanding of patient practices and of PP, health professionals’ perspectives also need to be explored. The second limitation is that our methodology was not designed to enumerate frequencies. Our research design was aimed at furthering our understanding of an under-theorized realm of patients’ experience. Future research could be devoted to enumerating frequencies, based on our findings. The third limitation is the study’s reliance on interviews as the sole source of data. We believe that future research should also explore how the three types of practices we identify are manifested in daily interactions with health professionals.

## Conclusions

Patients as partners are proactive in the care partnership, and this translates into three types of engagement practices. Learning practices allow them to acquire experiential knowledge about their health, as well as scientific information and technical know-how; assessment practices involve evaluating the quality of the partner relationship, as well as the health professional’s actions, recommendations, scientific knowledge, and technical know-how; and adaptation practices concern patient partners’ attempts to narrow the gap between what they need to ensure appropriate care and what health professionals provide.

These results directly affect health professionals’ practices, as well as informing those of administrators and decision-makers. By gaining a deeper understanding of patient partnership practices, health professionals are likely to feel less threatened and will be better able to establish trust-based relationships with their patients. Furthermore, administrators will be able to ensure that professionals in their institutions are better prepared to help patients obtain optimal care and services. Finally, at a policy level, a deeper understanding of patient partnership should help build an improved healthcare system where patients’ experiential knowledge and unique position as experts on living with their chronic illnesses allow them to be increasingly integrated and to have a greater role in ensuring optimal care.

A next step in exploring the notion of patient partnership should be to focus on the perspectives of healthcare professionals. How do they view patient partners, and what are the key components that enable them to establish constructive and trusting relationships with their patients? Only then will it be possible to gain a broader understanding of how productive interactions between healthcare professionals and patients can be strengthened at the clinical level.
